# Spatial-Temporal Analysis of PM_2.5_ and NO_2_ Concentrations Collected Using Low-Cost Sensors in Peñuelas, Puerto Rico †

**DOI:** 10.3390/s18124314

**Published:** 2018-12-07

**Authors:** Stephen Reece, Ron Williams, Maribel Colón, David Southgate, Evelyn Huertas, Marie O’Shea, Ariel Iglesias, Patricia Sheridan

**Affiliations:** 1 Oak Ridge National Laboratory, Oak Ridge, TN 37830, USA; 2 National Exposure Research Laboratory, Office of Research and Development, U.S. Environmental Protection Agency, Research Triangle Park, NC 27711, USA; Williams.ronald@epa.gov (R.W.); colon.maribel@epa.gov (M.C.); 3 DISUR, Inc., Ponce, PR 00716, USA; dsouthgate@disur.org; 4 U.S. Environmental Protection Agency, Region 2, Caribbean Environmental Protection Division, Guaynabo, PR 00968-8069, USA; Huertas.evelyn@epa.gov; 5 Region 2, U.S. Environmental Protection Agency, 290 Broadway, New York, NY 10007-1866, USA; Oshea.marie@epa.gov (M.O.); Iglesias.ariel@epa.gov (A.I.); 6 Region 2, U.S. Environmental Protection Agency, Edison, NJ 08837-3679, USA; Sheridan.patricia@epa.gov

**Keywords:** Low-cost sensors, air quality, citizen science, Puerto Rico

## Abstract

The U.S. Environmental Protection Agency (EPA) is involved in the discovery, evaluation, and application of low-cost air quality (AQ) sensors to support citizen scientists by directly engaging with them in the pursuit of community-based interests. The emergence of low-cost (<$2500) sensors have allowed a wide range of stakeholders to better understand local AQ conditions. Here we present results from the deployment of the EPA developed Citizen Science Air Monitor (CSAM) used to conduct approximately five months (October 2016–February 2017) of intensive AQ monitoring in an area of Puerto Rico (Tallaboa-Encarnación, Peñuelas) with little historical data on pollutant spatial variability. The CSAMs were constructed by combining low-cost particulate matter size fraction 2.5 micron (PM_2.5_) and nitrogen dioxide (NO_2_) sensors and distributed across eight locations with four collocated weather stations to measure local meteorological parameters. During this deployment 1 h average concentrations of PM_2.5_ and NO_2_ ranged between 0.3 to 33.6 µg/m^3^ and 1.3 to 50.6 ppb, respectively. Peak concentrations were observed for both PM_2.5_ and NO_2_ when conditions were dominated by coastal-originated winds. These results advanced the community’s understanding of pollutant concentrations and trends while improving our understanding of the limitations and necessary procedures to properly interpret measurements produced by low-cost sensors.

## 1. Introduction

The recent development of low-cost (<$2500) air quality (AQ) sensors has created new opportunities for individuals with various expertise to engage in AQ monitoring [1]. Traditionally, AQ monitoring has been limited to expensive regulatory monitors that require technical expertise to operate. However, low-cost sensors offer an affordable alternative with ease of use features to minimize user interaction and the ability to provide near real-time data. Since the emergence of these commercially available low-cost AQ sensors, they have been implemented in various applications to aid citizen scientists in environmental justice communities [2,3,4,5], promote environmental awareness by students and educators [5,6,7], supplement existing or establish novel AQ networks [5,8,9] and enhance monitoring capabilities of mobile applications [10,11].

The low-cost advantage of these AQ sensors provides the potential to deploy several sensors simultaneously to establish dense sensor networks [5,8,9] and/or as a multipollutant sensor pod [12]. Dense networks of low-cost sensors can be used to supplement existing regulatory networks or be deployed in remote areas entirely lacking AQ monitoring. These low-cost networks are able to provide real-time measurements across a small geographical area to detect diurnal trends and short-lived pollution events at a greater temporal and spatial resolution [13]. To gain additional insights about trends across pollutant types, multiple original equipment manufacturer (OEM) low-cost sensor units can be packaged with data acquisition software to create a multipollutant sensor pod. It has previously been shown that the ability to use low-cost sensors to measure the real-time relationship between various pollutants can be used to perform source attribution [9].

Despite the many advantages of low-cost sensors, the performance of these devices is still not well understood. Many efforts have been made by groups such as, the Joint Research Center (JRC), the South Coast Air Quality Management District (SCAQMD), and U.S. Environmental Protection Agency (EPA) to evaluate the performance of low-cost sensors and to provide a support framework for users [14,15,16,17]. Multiple studies have demonstrated the performance of these low-cost sensors compared to regulatory monitors varies across manufacturers and often between replicates of sensors [18,19,20]. The performance of many of these low-cost AQ sensors are impacted by environmental conditions to various degrees depending on the method of detection. Low-cost particulate matter (PM) sensors are largely dominated by light-scattering detection methods that have been shown to be influenced by relative humidity (RH) [20,21], concentration levels [22,23], aerosol type [23,24], and particle size distributions [23,25]. Low-cost gas-phase sensors mainly consist of electrochemical (EC), metal-oxide semiconductor (MOS), and photoionization detector (PIDs) based technologies that each have unique advantages and disadvantages [26]. Evaluation of these various low-cost gas-phase sensors have observed measurement discrepancies due to temperature, RH [13,18,27,28], and cross-sensitivity to non-target gases [28,29,30]. The inability to control these environmental parameters in real-world conditions often results in low-cost sensors performing worse in ambient environments compared to laboratory settings [18,23,29]. To overcome these limitations, deployment procedures and quality assurance (QA) protocols have been established to ensure the data collected were representative of reality [17].

Here we analyze spatial and temporal trends of PM_2.5_ (PM ≤ 2.5 microns) and nitrogen dioxide (NO_2_) concentrations using low-cost sensors in a micro-environment with relatively low ambient concentrations. Measurements were collected between October 2016–February 2017 using the EPA designed Citizen Science Air Monitor (CSAM) [31] by trained citizen scientists in an area of Puerto Rico identified as of interest by the community. The goal of this deployment was to use low-cost sensors to provide environmental awareness about local pollutant concentrations and to assess the performance of the low-cost sensors in a coastal environmental with elevated RH. The long-term performance of the low-cost sensors were assessed by collocating 2 CSAMs for the entire deployment. This study used concentrations of PM_2.5_ and NO_2_ to identify consistent trends between CSAM locations on a microscale level as a function of wind conditions and temporal variations.

## 2. Materials and Methods

### 2.1. Instrumentation

EPA developed a new version of the Citizen Science Air Monitor (CSAM) based on lessons learned from a previous deployment [2]. Briefly, the CSAMs consisted of low-cost (<$2500) OEM sensors, an Arduino Uno microprocessor for data acquisition, and an 8 GB secure digital (SD) card for data storage. For this deployment each CSAM was designed to collect real-time measurements at 5 min intervals of the particulate matter size fraction 2.5 micron (PM_2.5_), total volatile organic compounds (tVOCs), temperature (°C), and relative humidity (RH; % at °C). The CSAMs measured PM_2.5_ using an OPC-N2 Particle Monitor (Alphasense, Essex, United Kingdom) [32] and tVOCs with a Baseline Mocon piD-TECH sensor (MOCON Inc, Lyons, NY, United States) [33]. The OPC-N2 uses light particle counting to measure the concentration of suspended particles in the air sampled via an internal pump and has a detection limit of 0.1 μg/m^3^. The Baseline Mocon piD-TECH sensor uses a photoionization detector to measure a sum of all responding chemicals with a detection limit of 0.5 ppb isobutylene. Additionally, four CSAMs were equipped with a CairPol CairClip (Cairpol, Poissy, France) to measure real-time concentrations of nitrogen dioxide (NO_2_) [34]. The CairClip used a gas-specific inlet filter combined with dynamic air sampling in an integrated system to measure real-time NO_2_ concentrations with a detection limit of 1 ppb. During the deployment, four Vantage Vue weather stations (Davis Instruments, Hayward, CA, United States) were collocated with four CSAMs (CSAM 301, 304, 305, and 353/355) to provide meteorological parameters (rainfall, temperature, humidity, wind speed, and direction) every 30 min. Measurements collected from the tVOC sensors are not discussed in this manuscript pending additional investigation regarding sensor performance.

### 2.2. Deployment Area

**Figure 1:**
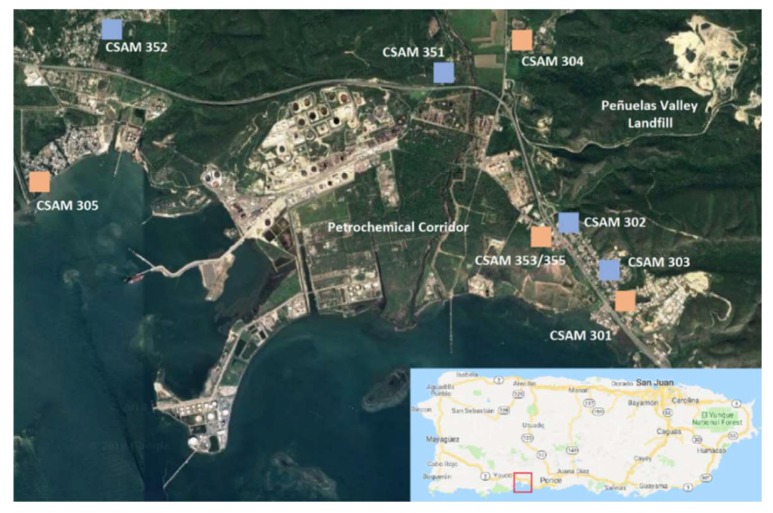
The deployment area in southern Puerto Rico is identified on the inset map with a red box. Approximate locations of CSAMs in the deployment area are identified with blue markers and locations with both a CSAM and weather station are identified with red markers. Image credit Google.

A citizen science led effort conducted between October 2016–February 2017 deployed nine CSAMs across eight ambient locations in a southern area of Puerto Rico (Tallaboa-Encarnación, Peñuelas) with little historical data available on pollutant spatial variability. These locations, shown in Figure 1, were within close proximity to residential communities, a major highway (Hwy 2), and various potential industrial sources. During the deployment two CSAMs were collocated to assess the long-term performance of the low-cost sensors. CSAMs were operated on approximately a weekly schedule where citizen scientists retrieved data and maintained all operations. Citizen scientists also recorded any visually observed pollution episodes or perceived smells during these routine site visits.

For clarity, the locations of the CSAMs and Vantage Vue weather stations are discussed in terms of the west, north, and southeast regions of the study area. The west region includes CSAMs 305, 352, and the West weather station. The north region includes CSAMs 304, 351, and the North weather station. The southeast region includes CSAMs 301, 302, 303, the collocated CSAMs 353/355 and the South and East weather stations.

### 2.3. Collocation Period

A 1-week collocation of CSAMs and weather stations was performed at the Environmental Quality Board (EQB) regulatory site in Ponce, Puerto Rico. While a period of sensor collocation versus regulatory monitoring during the deployment was a part of the study design, ultimately the local regulatory agency was unable to provide these data. The collocation period however identified improperly functioning sensors and established a median response per pollutant as a reference signal. Linear regressions were used to normalize each low-cost sensor and weather station to the reference signal to allow for spatial comparisons. Sensors that correlated poorly (R^2^ < 0.50) with the median reference signal were not included in the final analysis. This resulted in the exclusion of measurements from one PM_2.5_ sensor in the west (CSAM 305). Prior to deployment a multipoint calibration was performed on the NO_2_ sensors. A baseline check of each PM_2.5_ sensor was performed in a particle clean room as well as laboratory calibration of the RH, temperature, and tVOC sensors prior to deployment. Results of the collocation period are described in more detail in an earlier publication [35].

### 2.4. Data Analysis and Quality Assurance Procedures

To address quality assurance concerns about low-cost sensor data, detailed validation procedures were followed. The impact of these data validation procedures is discussed elsewhere [35]. Briefly, data were flagged for exclusion based on four criteria in the following order: human interference, non-responsive signal, influence of environmental conditions, and exceedance of the limit of detection. Data from the PM_2.5_ sensors were flagged when RH exceeded 90%. The removal of data collected under elevated RH conditions improved the precision between the PM_2.5_ sensors by 10.7% and accounted for between 2.5–21.8% of the dataset across CSAMs.

An EPA developed Microsoft Excel Macro Analysis Tool (MAT) was used for initial data analysis efforts [36]. The time interval, sampling rate, limit of detection (LOD), averaging period, and data completeness were required to be specified. The MAT data completeness constraint specified the percentage of data between the averaging period required to produce an average value. Once initialized, the MAT automatically time aligned the provided data and output averaged data with a visual representation. The validated 5 min data collected by the CSAMs were averaged with the MAT to 1 h and 24 h values using a data completeness of 80%.

The relative spatial variably between CSAM locations was explored using the Pearson coefficient (r) and coefficient of divergence (COD). The Pearson coefficient describes how well correlated two pollutant distributions are but not how similar. The COD describes the degree of homogeneity between two pollutant distributions on a scale of 0–1 defined as:

(1) $${\mathrm{COD}}_{\mathrm{fh}}=\;\sqrt{\frac{1}{n}\overset{n}{\underset{i=1}{\sum }}{\left(\frac{x_{\mathrm{if}}-x_{\mathrm{ih}}}{x_{\mathrm{if}}+x_{\mathrm{ih}}}\right)}^{2}}$$

where x_if_ and x_ih_ represent the average concentration at time i at locations f and h. The total number of concentrations compared in time-aligned pairwise fashion is represented by n. Homogenous pollutant distributions are described by low COD values (≤0.20) and values greater than 0.20 are considered dissimilar. The performance of the collocated PM_2.5_ and NO_2_ sensors (CSAM 353 and 355) was evaluated by looking at the change in correlation and precision during the deployment. The precision between sensors was calculated using the coefficient of variation (CV) described as:

(2) $$\mathrm{CV}=\;\frac{\sigma }{µ}$$

where σ represents the standard deviation between CSAM 353 and 355 and µ represents the mean concentration between CSAM 353 and 355. The CV describes the precision between two sensors using the ratio of the standard deviation and mean expressed as a percentage, with zero indicating perfect precision.

## 3. Results

### 3.1. Meteorological Conditions

Meteorological conditions were measured by four Vantage Vue weather stations at 30 min intervals. The weather stations were deployed alongside the CSAMs in a wind rose pattern to ensure results were representative of the entire deployment area. A summary of the monthly average temperature (°C), average RH (%), average wind speed (mph), and median wind direction (°) are reported in Table 1. Values for these parameters in November are not shown for the West weather station due to a delayed deployment. Temperatures were generally stable during the deployment period while RH decreased from November to February as Puerto Rico’s traditional rainy period concluded. Similarly, monthly average wind speed (WS) increased during the deployment at each weather station with winds originating predominantly from the east to south-southeast direction.

Active winds were defined as ≥ 2 mph. The East and South weather stations were the most active with 1 h average winds occurring 42% and 51.5% of the deployment respectively. At the East weather station 78.6% of the active winds approached from the east to southeast direction. The south weather station had the most active winds dominated by two directions. Winds between 2–3 mph arrived from the north-northeast to northeast direction (29.7% of active winds), while stronger winds between 3–6 mph originated from between the east and southeast directions (59.6% of active winds). The North weather station observed the least active winds (17.3% of deployment), typically approaching from the southeast to south-southeast direction at speeds between 2–4 mph. The West weather station observed active winds 27% of the deployment with 95.8% approaching from the southeast direction at WS predominantly greater than 6 mph. The strong winds observed by the East and West weather stations relative to the North and South weather stations were likely due to their proximity to the shoreline. Similarly, the reduced wind activity at the North weather station was likely due to being located more inland. The relationship between the local meteorological conditions and pollutant concentrations at these locations was explored here to better understand the micro-environmental conditions.

### 3.2. Long-Term CSAM Collocation

During the deployment, two CSAMs (353 and 355) in the southeast region were collocated to assess the change in correlation and precision over time between replicates of the PM_2.5_ and NO_2_ sensors. The correlation and precision were calculated on a weekly basis using normalized 5 min data. The weekly correlation between the replicate PM_2.5_ and NO_2_ sensors were also calculated using normalized 1 h average data to demonstrate the effect of increased averaging time on data quality. Previous evaluations of low-cost sensors have demonstrated that over time a sensor’s response can change due to a shift in baseline and degradation in the sensing mechanism of both light-scattering and gas-phase based sensors [20,37].

**Figure 2:**
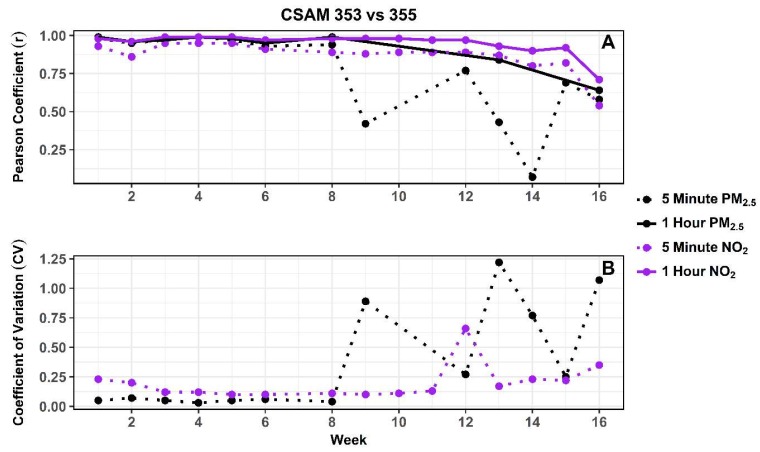
(**A**) Pearson coefficient (r) and (**B**) coefficient of variation (CV) calculated from 5 min (dashed line) and 1 h averaged (solid line) PM_2.5_ (black dots) and NO_2_ (purple dots) concentrations between October 2016–February 2017.

Change in correlation and precision over the duration of the deployment for the PM_2.5_ (black dots) and NO_2_ (purple dots) sensors are shown in Figure 2A,B. At the start of the deployment the PM_2.5_ sensors were initially better correlated (r: 0.98 > 0.93) and more precise (CV: 5% < 23%) compared to the NO_2_ sensors. Other studies have also observed greater inter-variability among gas-phase sensors compared to light-scattering sensors [15,17,38]. Figure 2A compares the correlation between 5 min (dashed line) and 1 h (solid line) average data from the PM_2.5_ and NO_2_ sensors. Averaging improved the correlation between the PM_2.5_ sensors from 0.75 to 0.92 and the NO_2_ sensors from 0.87 to 0.95. Figure 2A,B indicate that during the first 8 weeks the 5 min correlation between the PM_2.5_ sensors ranged between 0.93 and 0.99 and the precision between 3% and 7%. After week 8 the correlation (r = 0.07–0.77) and precision (CV = 25–122%) between the PM_2.5_ sensors were suddenly reduced. Further inspection revealed CSAM 353 began reporting a non-responsive PM_2.5_ signal during week 9 until being restarted to begin week 12. After week 12 CSAM 353 was non-responsive 96.2% of the remaining deployment compared to 0.9% of the deployment for CSAM 355. This intermittent PM_2.5_ response is highlighted in Figure 2A by the lack of 1 h average data compared to 5 min data available for weekly comparisons. The low correlation and precision between CSAM 353 and 355 indicates the response of CSAM 353’s PM_2.5_ sensor may have degraded or altered during the extended non-responsive period. To address this issue, all future analysis excluded PM_2.5_ measurements from CSAM 353 after week 8 (11:00 AM, 22 December 2016).

During the first 6 weeks the 5 min correlation between NO_2_ sensors ranged between 0.86 and 0.95 while the precision improved from 23% to 10%. This initial improvement in precision was potentially due to the electrochemical sensors stabilizing to new environmental conditions. During weeks 8 to 11 the 5 min correlation (r = 0.88–0.89) and precision (CV = 10–13%) were stable. Figure 2A,B show the NO_2_ sensors maintained correlation (r = 0.89) during week 12, but for unexplained reasons a decrease in precision was observed (CV = 66%). During weeks 13 to 16 the correlation (r = 0.87–0.54) and precision (CV = 17–35%) between NO_2_ sensors gradually decreased. This gradual increase in variability between collocated sensor replicates was likely due to potential degradation of the sensor transducer [20,37]. The median value of CSAM 353 and 355, referred to as CSAM353/355, was used for all following temporal and spatial pollutant analysis reported here. During periods when only one of the two collocated CSAMs were reporting, then the value of the functioning CSAM was used for comparisons.

### 3.3. 1 h Average Pollutant Concentrations

**Figure 3:**
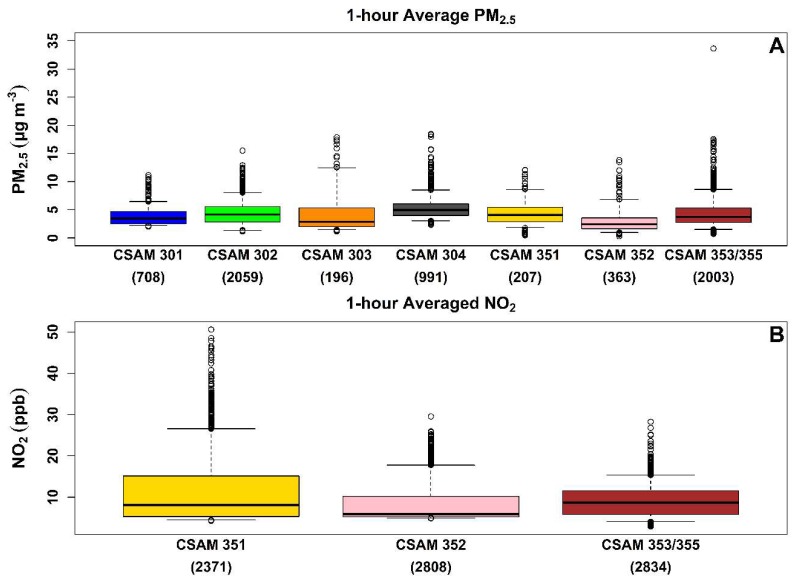
The 1 h average PM_2.5_ (**A**) and NO_2_ (**B**) concentrations collected during the deployment for each CSAM location. The box represents the interquartile range of 25th and 75th percentile and the whiskers indicate the 5th and 95th percentile. The horizontal line in each box is the median concentration. The x-axis displays the number of 1 h average data points measured at each CSAM location.

The normalized 5 min PM_2.5_ and NO_2_ measurements were averaged on an hourly basis using the MAT [36] with a data completeness of 80%. Distributions of 1 h average PM_2.5_ and NO_2_ concentrations are shown in Figure 3A,B. The box represents the interquartile range of 25th and 75th percentile and the whiskers indicate the 5th and 95th percentile. The horizontal line in each box is the median concentration. The number of 1 h average data points measured at each CSAM location is displayed along the x-axis. The variation in data points collected between CSAM locations was the result of sensor failure and/or interference of environmental conditions. This resulted in a data completeness between 8.6% and 80.0% for the PM_2.5_ sensors and between 97.4% and 99.7% for the NO_2_ sensors. Sensor performance and quality assurance procedures are discussed in more detail in a previous publication [35].

Median 1 h average PM_2.5_ concentrations ranged between 2.4 and 4.9 µg/m^3^ during the deployment. The lowest median 1 h average PM_2.5_ concentration was observed by the western most location (CSAM 352), in a residential community downwind of Hwy 2. The northern mainland locations (CSAM 304 and 351) reported the highest 1 h average PM_2.5_ concentrations (4.1–4.9 µg/m^3^). The largest median PM_2.5_ concentration was reported by CSAM 304 located in a northern residential community two kilometers (km) west of the Peñuelas Valley Landfill. The median 1 h average PM_2.5_ concentrations in the southeast, with the exception of CSAM 302, were low (2.9–3.7 µg/m^3^) relative to the northern mainland locations. The median PM_2.5_ concentration observed at CSAM 302 (4.1 µg/m^3^) was more elevated than other southeast locations. The range of median PM_2.5_ concentrations observed during this deployment overlap with mean PM_2.5_ concentrations (4.20–4.84 µg/m^3^) reported across various rural areas in Puerto Rico for 8 weeks between 14 March 2005 to 6 May 2005 using a portable MET One 531 in a separate air quality study [39]. An additional study operated a MET One Neighborhood monitor at the University of Puerto Rico’s Rio Piedras campus from 22 November 2017 to 22 December 2017 and observed an average 1 h PM_2.5_ concentration of 3.5 ± 2.3 µg/m^3^ [40]. The MET One is a low-cost PM sensor that uses light-scattering detection similar to the PM sensor deployed in this study. We reference these studies here only to provide a general review of the localize air quality conditions in other areas of Puerto Rico. The mean PM_2.5_ concentrations (5.03–6.32 µg/m^3^) reported by the MET One in urban areas of Puerto Rico were only slightly greater than the median 1 h average PM_2.5_ concentrations observed in this study [39].

The maximum 1 h average median PM_2.5_ concentration (33.6 µg/m^3^) observed during the deployment was at the South weather station (CSAM 353/355). This 1 h average concentration was examined beyond the required quality assurance procedures to ensure the validity of this data point. Unfortunately, during this period CSAM 353 was non-responsive, preventing the comparison of the collocated CSAMs. Evaluation of normalized 5 min data during this period on 23 December 2016 reveals a bimodal PM_2.5_ event. The first event starts at approximately 8:00 AM and peaks at 141.6 µg/m^3^ by 8:25 AM. A second smaller event begins at 8:40 AM and peaks at 12.0 µg/m^3^ by 9:00 AM. Further inspection of the normalized 5 min data during this period identified a similar bimodal PM_2.5_ event of lesser magnitude at a nearby location across from Hwy 2 (CSAM 302). At this location, PM_2.5_ concentrations suddenly increased from 5.8 to 26.1 µg/m^3^ at 9:00 AM, followed by a second event at 9:20 AM that peaked at 13.3 µg/m^3^ at 9:30 AM. The response of the PM_2.5_ sensor to this event (peak followed by gradual decline) in addition to the observation of a similar event of a lesser magnitude at a nearby location indicates this data point was suggestive of a true PM_2.5_ event. The observed variation in PM_2.5_ concentrations between CSAMs was indicative of micro-environmental conditions that were then explored temporally and spatially.

The 1 h average median NO_2_ concentrations shown in Figure 3B ranged between 5.9 and 8.7 ppb. The lowest 1 h average median NO_2_ concentration was observed at the west location (CSAM 352), similar to the PM_2.5_ concentrations. The west location was positioned the farthest from Hwy 2 followed by the north (~100 m) and southeast (~15 m) locations. The 1 h average median NO_2_ concentrations observed at these locations decreased as a function of the distance from Hwy 2. The north location (CSAM 351) consistently observed higher 1 h average NO_2_ concentrations despite being located farther from Hwy 2 and having a lower median NO_2_ concentration than the southeast location (CSAM 353/355). This is shown in Figure 3B by the wider distribution skewed towards higher NO_2_ concentrations at the north location. The maximum 1 h NO_2_ concentration reported by EQB monitoring stations during our deployment period was 159 ppb (Caguas, PR) and 195 ppb (Guaynabo, PR) in 2016 and 104 ppb (Caguas, PR) and 28 ppb (Guaynabo, PR) in 2017. The maximum 1 h NO_2_ concentration of 50.6 ppb observed by the north location was still lower than the NAAQS 1 h NO_2_ concentration of 100 ppb.

### 3.4. Spatial Analysis of 1 h Average PM_2.5_ and NO_2_ Concentrations

**Figure 4:**
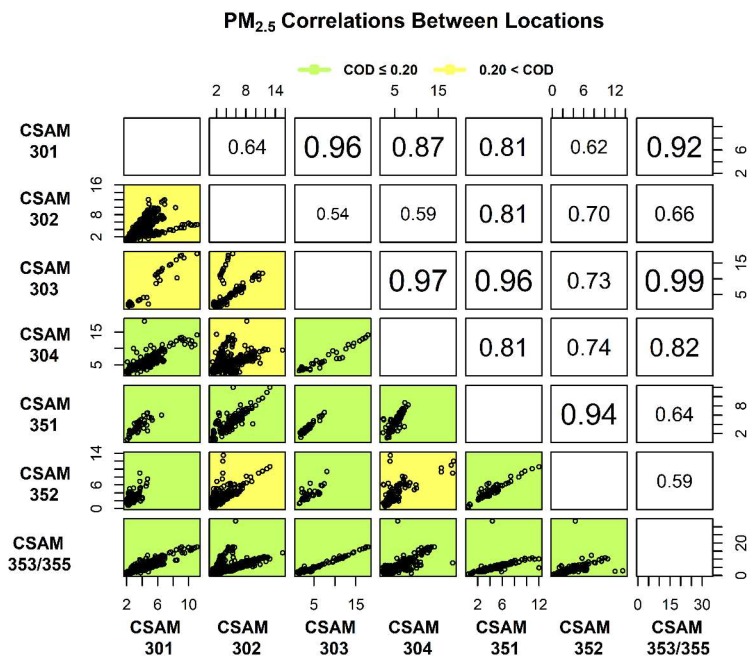
1 h average PM_2.5_ concentrations were time-aligned in pairwise manner to calculate the Person coefficient (r) and Coefficient of Divergence (COD) between each location. The r values are numerically reported in the upper right and increase in font size with improved correlation. Scatter plots in the lower left visually display the correlations and backgrounds are colored either green (homogenous) or yellow (heterogeneous) to represent the COD values.

The spatial comparison of 1 h average PM_2.5_ and NO_2_ distributions between locations were analyzed using COD and r values calculated in a time-aligned pairwise fashion and shown in Figure 3 and Figure 4. Correlations between CSAM locations are numerically reported in the upper right and visually displayed as scatter plots in the lower left. The correlations reported in Figure 3 and Figure 4 increases in font size with improved correlation. The background of the scatter plots in the lower left are color coded either green (COD ≤ 0.20) or yellow (COD > 0.20) to indicate homogeneity. To account for measurement uncertainty in the calculation of COD and r values, the PM_2.5_ and NO_2_ concentrations measured by the collocated CSAMs were compared. The spatial comparison between collocated CSAMs had COD and r values of 0.03 and 0.99 for PM_2.5_ and 0.17 and 0.70 for NO_2_. The higher COD and lower r values of the collocated NO_2_ sensors compared to the PM_2.5_ sensors were reflective of the greater measurement uncertainty of the NO_2_ sensors. These values provided a baseline for comparisons between the other CSAM locations, with r ≥ 0.80 indicating correlation between PM_2.5_ sensors and r ≥ 0.60 for NO_2_ sensors.

The spatial comparison between PM_2.5_ distributions across CSAM locations is shown in Figure 4. The correlations and COD values shown in Figure 4 were also recalculated while excluding the previously discussed elevated concentration observed at the South weather station to determine the impact on analysis. The removal of this data point improved the correlation between CSAMs 351 and 353/355 (r = 0.64–0.92) but did not significantly affect any other correlations or COD values. This elevated concentration was therefore excluded. The PM_2.5_ distributions in the southeast, excluding CSAM 302, were well correlated (r = 0.92–0.99). Comparisons between the southeast and north regions were slightly less correlated (r = 0.81–0.97) but indicated similar responses. The correlation between CSAM locations in the north (r = 0.81) was less than in the southeast, possibly due to differences in wind activity. As previously mentioned, the wind activity in the southeast region on the coast was more active (42–51.5%) than in the mainland to the north (17.3%).

The 1 h average PM_2.5_ concentrations reported by CSAM 351 in the north were similar (COD = 0.08–0.18) and correlated (r = 0.81–0.96) to all locations. This agreement with the other locations was likely due to either CSAM 351’s relatively central location or low data completeness (10.1%). The western most location and the elevated location in the southeast (CSAM 302) were uncorrelated with all locations except CSAM 351. Figure 4 shows the relatively low PM_2.5_ distribution in the west was spatially unique (COD = 0.24–0.32) when compared to the 2 locations with the highest median 1 h average PM_2.5_ concentrations (CSAMs 302 and 304). Similarly, CSAM 302 was the most spatially unique location with the majority of comparisons resulting in COD values > 0.20, despite other CSAMs located nearby in the southeast region. The lack of correlation and spatial homogeneity between CSAM 302 and the other southeast locations in conjunction with the relatively higher median 1 h average PM_2.5_ concentration (Figure 3A) was suggestive of a local source of PM_2.5_ unique to this location. Alternatively, the lack of correlation and low PM_2.5_ concentrations reported by the west location compared to other CSAMs was suggestive this location was more representative of background conditions. To maximize the data available for comparisons, the three CSAM locations that collected the most 1 h average PM_2.5_ measurements (CSAMs 302, 304, and 353/355) were used for additional spatial and temporal analysis [35].

**Figure 5:**
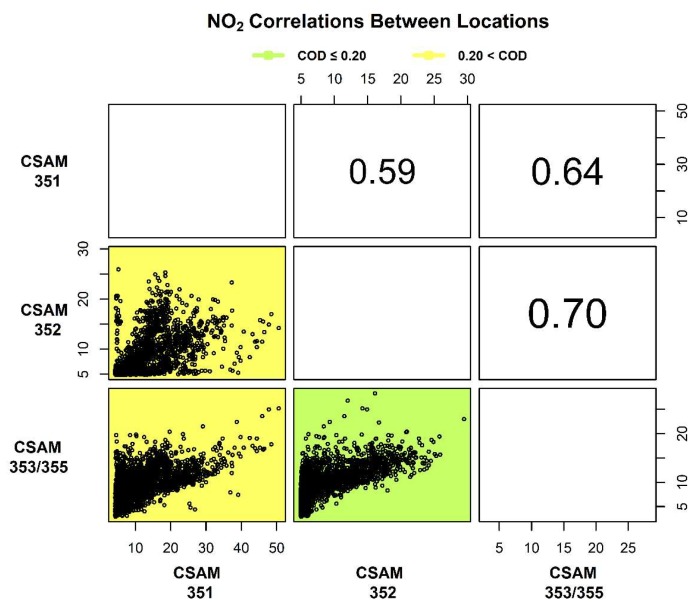
1 h average NO_2_ concentrations were time-aligned in pairwise manner to calculate the Person coefficient (r) and Coefficient of Divergence (COD) between each location. The r values are numerically reported in the upper right and increase in font size with improved correlation. Scatter plots in the lower left visually display the correlations and backgrounds are colored either green (homogenous) or yellow (heterogeneous) to represent the COD values.

It was previously shown the median 1 h average NO_2_ concentrations decreased across regions (southeast-west), as a function of distance from Hwy 2. Spatial analysis of the 1 h average NO_2_ concentrations in Figure 5 shows the southeast and west locations were equivalently correlated (r = 0.70) and spatially homogeneous (COD = 0.17) as the collocated NO_2_ sensors. The similar response from the southeast and west locations likely indicated a similar near-road source with the distance from the road influencing the difference in median 1 h average concentrations (Figure 3B). The lower correlation (r = 0.59–0.64) and dissimilarity (COD = 0.22–0.23) of the NO_2_ concentrations observed at the north location, in conjunction with higher maximum concentrations (Figure 3B), indicated a unique response potentially due to an additional source of NO_2_. To further explore these spatial relationships, wind speed and direction were examined to isolate conditions when distribution of concentrations were correlated or similar.

### 3.5. Spatial Analysis of PM_2.5_ and NO_2_ Concentrations as a Function of Wind Conditions

**Figure 6:**
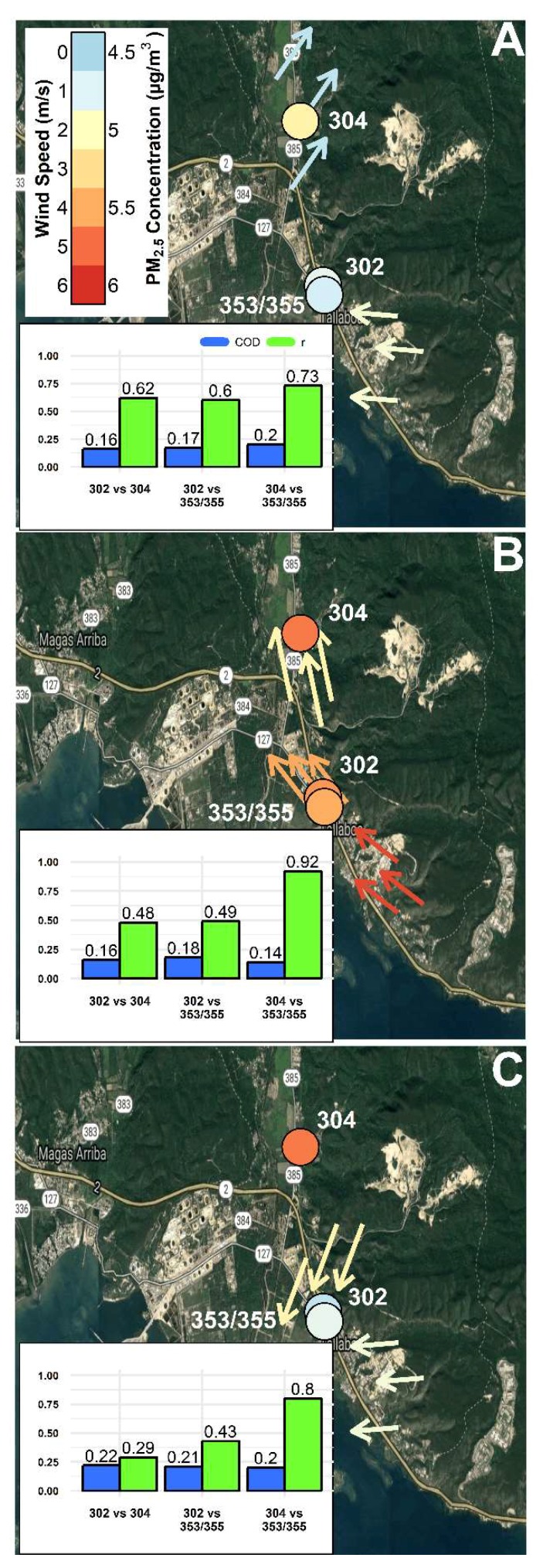
The change in 1 h average PM_2.5_ concentrations across 3 CSAM locations (CSAMs 302, 304, and 353/355) were explored as a function of the following wind conditions observed at the South weather station: (**A**) inactive, (**B**) ocean-originated, and (**C**) mainland-originated. COD (blue bars) and r (green bars) values were recalculated in a pairwise fashion between locations for each wind condition and displayed as a bar chart in the bottom left of Figure 6A–C. Wind conditions at the South, East, and North weather stations are depicted by arrows indicating the median wind direction as a function of the conditions at the South weather station and colored by WS. Each CSAM location in Figure 6A–C are similarly colored based on pollutant concentrations.

**Figure 7:**
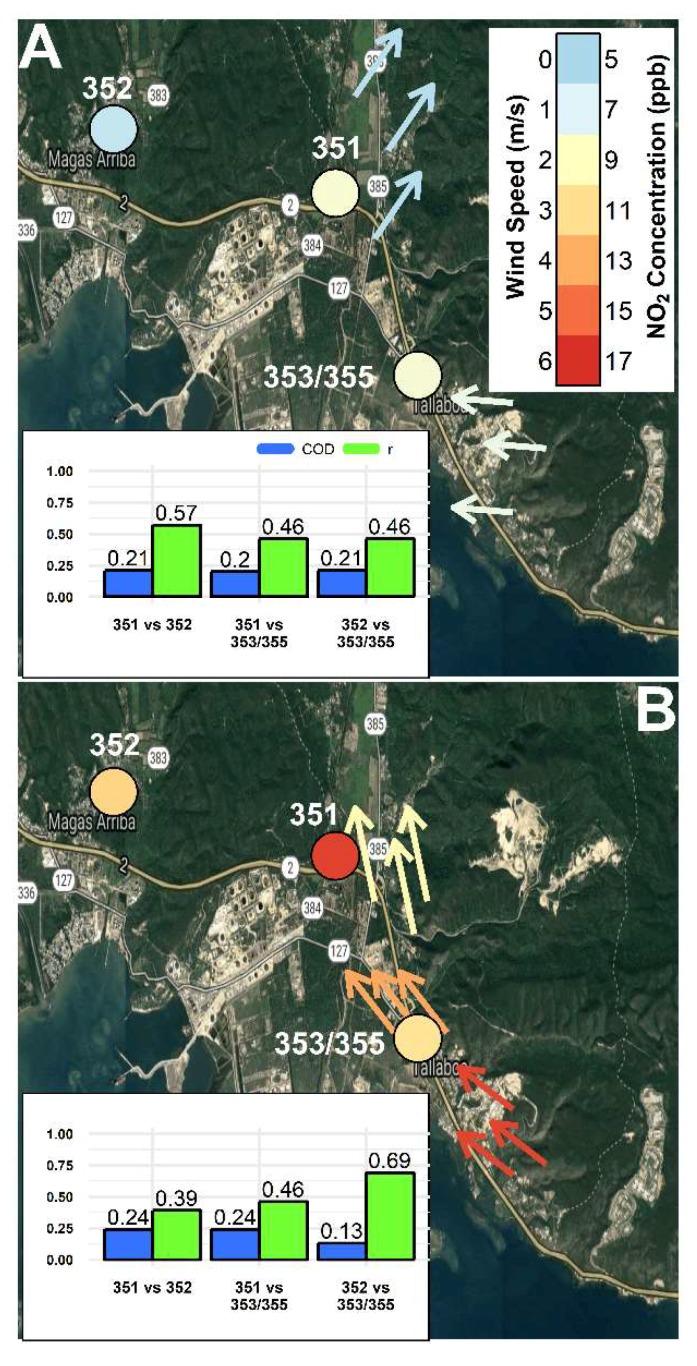

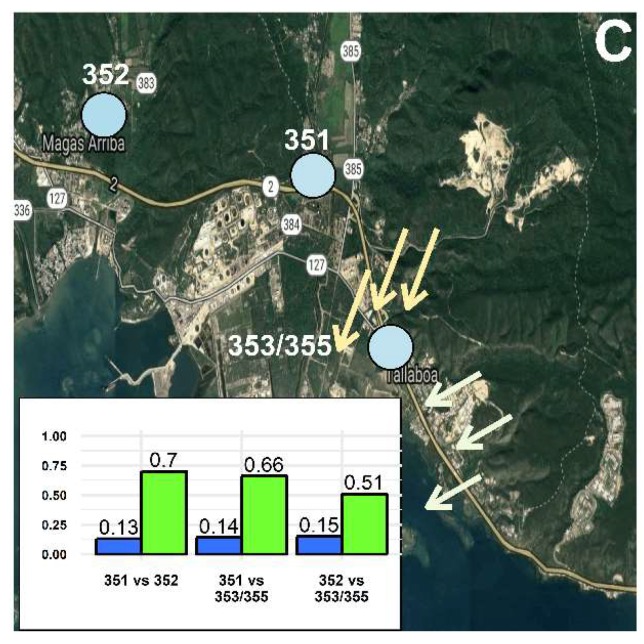
The change in 1 h average NO_2_ concentrations across 3 CSAM locations (CSAMs 351, 352, and 353/355) were explored as a function of the following wind conditions observed at the South weather station: inactive (**A**), ocean-originated (**B**), and mainland-originated (**C**). COD (blue bars) and r (green bars) values were recalculated in a pairwise fashion between locations for each wind condition and displayed as a bar chart in the bottom left of Figure 7A–C. Wind conditions at the South, East, and North weather stations are depicted by arrows indicating the median wind direction as a function of the conditions at the South weather station and colored by WS. Each CSAM location in Figure 7A–C are similarly colored based on pollutant concentrations.

The spatial relationship of pollutants between CSAM locations was further explored by examining the effect of changing wind conditions. Using the South weather station as a reference, 1 h average meteorological data were used to identify the following 3 wind conditions: inactive winds (Figure 6A and Figure 7A), costal-originated winds (Figure 6B and Figure 7B), and mainland-originated winds (Figure 6C and Figure 7C). Periods of inactive winds occurred 48.5% of the deployment at the South weather station. Figure 6A and Figure 7A shows during periods of inactive winds at the South weather station the North and East weather stations similarly observed weak (<2 mph) winds from the SW and E directions, respectfully. Figure 6B and Figure 7B shows the ocean-originated winds (30.7% of deployment) were characterized by strong WS approaching from the east-southeast to south-southeast direction that weaken the farther inland. Figure 6C and Figure 7C shows as winds transitioned to a mainland-originated wind approaching from the north-northeast (15.3% of deployment), the winds in the north diminish and the East weather station shifted back to a weak easterly direction, similar to the period of inactive winds (Figure 6A and Figure 7A). This demonstrated the coastal and mainland winds were only active during the period of ocean-originated winds (Figure 6B and Figure 7B). The 1 h averaged PM_2.5_ and NO_2_ concentrations from 3 locations were time-aligned and binned by the 3 defined wind conditions. COD (blue bars) and r (green bars) values were recalculated in a pairwise fashion between locations for each wind condition and displayed as a bar chart in the bottom left of Figure 6A–C and Figure 7A–C. Wind conditions at the South, East, and North weather stations are depicted by arrows indicating the median wind direction as a function of the conditions at the South weather station and colored by WS. Each CSAM location in Figure 6A–C and Figure 7A–C are similarly colored based on pollutant concentrations.

In Figure 6A–C, the three CSAM locations compared were located at the North weather station (CSAM 304), South weather station (CSAM 353/355), and in a southern residential community (CSAM 302). The South weather station was a near-road location (~15 m from Hwy 2) located at a local public school. The southern residential community (CSAM 302) was located approximately 0.5 km adjacent to the South weather station on the opposite side of Hwy 2 at a 30 m elevation. The North weather station was located approximately ~2.3 km north of the South weather station in a residential community 2 km west of the Peñuelas Valley Landfill.

The 1 h average PM_2.5_ concentrations were spatially homogeneous (COD = 0.16–0.20) and similarly correlated (r = 0.60–0.73) between all locations during the period of inactive winds shown in Figure 6A. As coastal-originated winds began to approach, PM_2.5_ concentrations peaked across all 3 locations and maintained spatial homogeneity (COD = 0.14–0.18). Although PM_2.5_ distributions were similar across all 3 locations, only the north and southern near-road location were correlated (r = 0.92). As winds transitioned from coastal to mainland-originated, the north and southern near-road locations remain correlated (r = 0.80). During all three wind conditions (Figure 6A–C) the PM_2.5_ concentrations were greater in the north relative to the south, similar to the PM_2.5_ distributions shown in Figure 3A. Figure 6A–C indicate similar responses between the north and southern near-road locations as correlations improved with increased wind activity. The southern residential location was only similarly correlated during periods of inactive winds and relatively low PM_2.5_ concentrations. A possible explanation could be the southern residential location was on the opposite side of Hwy 2, upwind of potential near-road PM_2.5_ sources. This location was therefore possibly more representative of local background concentrations. Other near-road studies have utilized similar site selections with respect to wind direction to differentiate between background and on-road pollution [41,42].

In Figure 7A–C 3 near-road CSAM locations in the southeast (CSAM 353/355), north (CSAM 351), and west (CSAM 352) were compared during the previously defined wind conditions. The 1 h average NO_2_ concentrations shown in Figure 7A were more variable (COD = 0.20–0.21) and uncorrelated (r = 0.46–0.57) during inactive winds. Figure 7A shows the farthest near-road location in the west observed lower NO_2_ concentrations. Similar to the PM_2.5_ concentrations, NO_2_ concentrations peaked at every location during coastal-originated wind conditions. During coastal-originated wind conditions, shown in Figure 7B, the west and southeast near-road locations were similar (COD = 0.13) and correlated (r = 0.69) indicating the influence of a similar near-road source (Hwy 2). The north near-road location was dissimilar (COD = 0.24) and uncorrelated (r = 0.39–0.46) compared to the west and southeast locations. The north near-road location also observed relatively higher NO_2_ concentrations than the southeast location despite a farther distance from Hwy 2. This spatially unique location with relatively higher concentrations could be indicative of an additional source to the south-southeast of the north location shown in Figure 7B. When wind conditions changed to being mainland-originated, minimum NO_2_ concentrations were observed at all 3 locations probably due to the sites no longer being downwind of the near-road sources. Comparisons between distributions of NO_2_ concentrations indicated homogeneity (COD = 0.13–0.15) and correlations varied as a function of distance between the compared locations as shown in the bar plot inset in Figure 7C. The wind condition shown in Figure 7C was likely representative of local background NO_2_ concentrations. The analysis of Figure 7A–C demonstrated data from the study being useful to to spatially compare pollutant concentrations as a function of wind conditions and identify isolated locations and/or periods with elevated concentrations.

### 3.6. Temporal Comparisons

**Figure 8:**
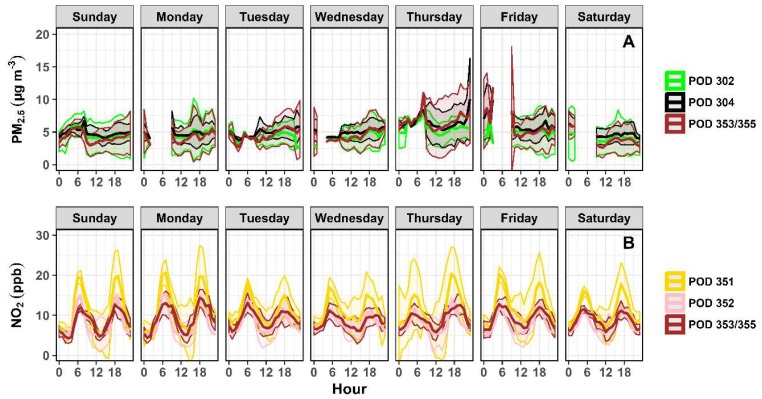
The 1 h average PM_2.5_ (**A**) and NO_2_ (**B**) time-aligned concentrations were binned hourly on a weekly basis to explore the spatial relationship between CSAM locations as a function of time. Shading indicates the standard deviation binned hourly. Gaps in PM_2.5_ concentrations during certain periods indicate either 1 or more sensors were non-responsive.

To explore the weekly variation between CSAM locations, 1 h average PM_2.5_ and NO_2_ distributions were time-aligned and binned hourly. Any data gaps in Figure 8 were the result of one or more non-functioning sensors during the period. The spatial variation between CSAM locations was compared using daily average r and COD values calculated in a pairwise fashion. Temporal analysis allowed concentrations to be compared on an hourly basis to identify diurnal trends and pollution events independent of wind conditions.

The weekly variation in PM_2.5_ concentrations between CSAMs 302, 304, and 353/355 is shown in Figure 8A. Across all three locations, PM_2.5_ concentrations were the lowest on the weekend (3.6–4.7 µg/m^3^) and continuously increased during the week until peaking on Thursday (5.4–6.5 µg/m^3^). Similar to earlier observations, concentrations in the north (CSAM 304) were relatively higher compared to the southeastern locations. The southeastern near-road location (CSAM 353/355) observed the largest difference between average PM_2.5_ concentrations on the weekend (3.9 µg/m^3^) compared to during the week (5.3 µg/m^3^). The adjacent location across Hwy 2 observed the least difference between weekend (4.3 µg/m^3^) and weekday (4.8 µg/m^3^) PM_2.5_ concentrations. A possible explanation for the relatively lower weekend PM_2.5_ concentrations was reduced on-road vehicles. This would also explain why the largest change in PM_2.5_ concentrations were observed at the near-road location. During the week the north and southeastern near-road locations were correlated everyday (r = 0.84–0.95), with exception to Friday (r = 0.64) when none of the locations were correlated. This maintained correlation between locations was likely due PM_2.5_ concentrations being dominated by on-road vehicles during the week. It was previously shown by Figure 6A–C that the relationship between the north and southeastern near-road locations improved when sites were downwind of Hwy 2. Similar to the spatial analysis of PM_2.5_ concentrations above, the southeastern residential community was only correlated (r = 0.91–0.95) and similar (COD = 0.14–0.18) to the adjacent near-road site during low PM_2.5_ concentrations on the weekend. This further supported the belief that the north and southeastern near-road locations were dominated by on-road PM_2.5_ sources not detected by the location upwind of Hwy 2 (CSAM 302).

Temporal analysis of NO_2_ concentrations, shown in Figure 8B, revealed a bimodal distribution of NO_2_ concentrations at each near-road location occurring on a daily basis. NO_2_ concentrations peaked at each location at approximately 6:00 AM and 6:00 PM. Other near-road studies have observed similar trends in NO_2_ concentrations indicating this response was likely a function of morning and evening on-road commuters. Daily average NO_2_ concentrations were consistent on a weekly basis, with higher concentrations observed at the north near-road location (10.6 ± 0.7 ppb) compared to the west (7.9 ± 0.2 ppb) and southeast (8.8 ± 0.5 ppb) locations. Spatial analysis demonstrated the west (CSAM 352) and southeast (CSAM 353/355) near-road locations were similar (COD = 0.15–0.18) every day and correlated (r = 0.63–0.76) every day, except Sunday. The relationship between the west and southeast locations, despite distance, indicated NO_2_ concentrations were likely being dominated by a common source such as Hwy 2. Figure 7A–C revealed the correlation and similarity between the southeast and west locations improved when active winds were blowing downwind or upwind of Hwy 2. The north location on average was equivalently similar to both the west (COD = 0.20 ± 0.03) and southeast (COD = 0.20 ± 0.01) locations as was shown in Figure 7A–C. However, the north location on average was more correlated with the west (r = 0.70 ± 0.08) than the southeast (r = 0.63 ± 0.07). This same relationship between locations was observed during non-coastal-originated source winds (Figure 7A,C). This was expected as daily wind activity was dominated by non-coastal-originated winds. Although the southeast location was closer to the north than the west location, correlations were lower and NO_2_ distributions were more dissimilar, indicating a potential additional source of NO_2_ unique to the north near-road location. This belief was supported by the spatial analysis that demonstrated the north near-road location observed the largest delta change in NO_2_ concentrations (7.8 ppb) and became uncorrelated (r = 0.39–0.46) as coastal-originated winds approached from upwind of the near-road source.

## 4. Discussion

In this study citizen scientists deployed and maintained low-cost AQ sensors for an extended period which provided the basis for extensive data analyses on the spatial and temporal variability of select air pollutants. Two types of low-cost sensors were collocated for an extended period to evaluate their performance in a harsh costal environment. The extended collocation provided observations that a sudden change in response between some PM_2.5_ sensors had occurred that would have been otherwise undetected. This demonstrates the need to incorporate routine QA checks during deployment periods to verify measurements. Experimental designs that include collocation at the beginning and end of a deployment are able to account for natural degradation in sensor performance but are unable to identify real-time sensor failure in situations where a sensor reports values unreflective of reality. Additionally, it has been shown that sensor performance is a function of localized environmental conditions and collocations performed outside the deployment area are typically not useful as part of the development of a correction algorithm [18]. Studies have attempted to address this issue by deploying AQ instruments with higher precision and accuracy that have been calibrated against a reference instruments alongside the low-cost sensors and periodically rotating them about the low-cost network [43].

An advantage of real-time measurements was shown to be the ability to establish diurnal trends across pollutants. This allowed specific days and hours when concentration peaked to be identified to contribute to communities understanding of pollutants. Although these low-cost sensors were not free of issues (low data completeness, environmental influences, poor agreement) rigorous QA guidelines allowed for the validation of an extensive data set. A pending manuscript details the QA process for verifying these data and examines the success and failures of the deployment from a citizen science perspective. These low-cost sensors demonstrated the ability to advance a community’s understanding of pollutant concentrations and trends while improving our understanding of the limitations and necessary procedures to properly interpret measurements produced by low-cost sensors.

## 5. Conclusions

1 h average concentrations of PM_2.5_ (0.3–33.6 µg/m3) and NO_2_ (1.3–50.6 ppb) were relatively low across the deployment area. The median 1 h average PM_2.5_ concentrations were greater in the north region and lower in the west region. The median 1 h average NO_2_ concentrations varied as a function of distance from Hwy 2. Despite the low range of observed PM_2.5_ and NO_2_ concentrations, relationship between CSAM locations identified uncorrelated and heterogeneous pollutant distributions. The relationships established between CSAM locations were further explored as a function of wind conditions using the Vantage Vue weather stations. Wind conditions across the island were predominantly calm (WS < 2 mph), with the majority of active winds originating from the coastal direction. PM_2.5_ concentrations peaked when conditions were dominated by coastal-originated winds. Correlations between PM_2.5_ concentrations in the north (CSAM 304) and southeastern residential community (CSAM 353/355) improved with increased wind activity indicating a similar response. PM_2.5_ concentrations at the neighborhood location (CSAM 302) upwind of Hwy 2 became less correlated with increasing wind activity and increasing PM_2.5_ concentrations. Similar to PM_2.5_ concentrations, NO_2_ concentrations peaked as coastal-originated winds approached with NO_2_ sensors located downwind of Hwy 2. The lowest NO_2_ concentrations were observed at all locations as winds shifted to mainland-originated winds and the NO_2_ sensors were upwind of the near-road source. During the mainland-originated regime, correlations between NO_2_ concentrations varied as a function of distance between CSAM locations. When the NO_2_ sensors were downwind of Hwy 2 the north location observed the largest increase in concentration and became less similar and uncorrelated, indicating a potential additional local source of NO_2_. Pollutant distributions were then analyzed temporally to explore how concentrations vary on a weekly basis. PM_2.5_ concentrations were lowest on the weekends and gradually increased during the week until peaking on Thursday. PM_2.5_ concentrations in the north (CSAM 304) and southeastern residential community (CSAM 353/355) were correlated every day of the week, with exception to Friday, while the neighborhood location (CSAM 302) was only correlated on the weekend when concentrations were lower. NO_2_ concentrations consistently peaked at 6:00 AM and PM daily likely indicating a response to morning and evening on-road commuters. Similar to the trends observed during the spatial analysis, correlations were lower and NO_2_ distributions were more dissimilar at the north near-road location, indicating a potential additional source of NO_2_.

## Figures and Tables

**Table 1:** The monthly average temperature (°C), relative humidity (%), wind speed (mph) and 1 h median wind direction (°) for the East, North, South, and West weather stations. “UN” indicates measurements for the period were unavailable.

Weather Station	Meteorological Condition	November	December	January	February
East (CSAM 301)	Temperature (°C)	26.8	26.8	25.2	26.5
	RH (%)	79	71	70	69
	Wind Speed (mph)	2.3	2.7	2.6	3.7
	Wind Direction (°)	ESE (115.0)	ESE (115.0)	ESE (115.0)	ESE (115.0)
North (CSAM 304)	Temperature (°C)	25.5	25.3	23.9	25.2
	RH (%)	87	80	78	76
	Wind Speed (mph)	0.5	0.7	0.8	1.4
	Wind Direction (°)	E (93.5)	SE (138.5)	SSE (161.0)	SSE (161.0)
South (CSAM 353/355)	Temperature (°C)	26.0	26.4	25.1	26.2
	RH (%)	79	73	72	71
	Wind Speed (mph)	1.8	2.3	2.3	3.0
	Wind Direction (°)	E (79.7)	ESE (111.8)	ESE (111.8)	SE (127.8)
West (CSAM 305)	Temperature (°C)	UN	24.8	23.8	24.8
	RH (%)	UN	80	79	80
	Wind Speed (mph)	UN	1.9	1.5	3.5
	Wind Direction (°)	UN	127.0 (SE)	127.0 (SE)	127.0 (SE)
